# Assessment of radiation pneumonitis and predictive factors in patients with locally advanced non-small cell lung cancer treated with chemoradiotherapy

**DOI:** 10.2340/1651-226X.2024.40576

**Published:** 2024-10-16

**Authors:** Kerstin Gunnarsson, Louise Mövik, Niclas Pettersson, Anna Bäck, Jan Nyman, Andreas Hallqvist

**Affiliations:** a Department of Oncology, Institute of Clinical Sciences, Sahlgrenska Academy, University of Gothenburg, Sahlgrenska University Hospital, Gothenburg, Sweden; b Department of Medical Radiation Sciences, Institute of Clinical Sciences, Sahlgrenska Academy, University of Gothenburg, Sahlgrenska University Hospital, Gothenburg, Sweden

**Keywords:** Radiation pneumonitis, non-small cell lung cancer, chemoradiotherapy, predictive factors, recovery rate

## Abstract

**Purpose:**

Radiation pneumonitis (RP) is a dose-limiting toxicity associated with increased mortality for patients with non-small cell lung cancer (NSCLC) treated with chemoradiotherapy (CRT). This study aims to assess the incidence of symptomatic RP (grade 2–5), rate of recovery and associated predictive factors.

**Material and methods:**

We performed a retrospective population-based study including 602 patients with NSCLC who were treated with CRT between 2002 and 2016. RP and rate of recovery were analysed using Common Terminology Criteria for Adverse Events version 4.0. Stepwise logistic regression was performed to analyse potential predictive factors for the two endpoints RP grade ≥ 2 and RP grade ≥ 3.

**Results:**

A total of 136 (23%) patients developed symptomatic RP and 37 (6%) developed RP grade ≥ 3. A total of 67 (71%) recovered, whereas the remaining 27 (29%), with the major proportion of patients belonging to the RP grade ≥ 3 group, suffered from prevailing sequelae. On multivariable analysis, the selected model for predicting RP grade ≥ 2 included the factors V_20_, smoking status, average fractions per week and chemotherapy agent. V_20_ and age were selected factors for RP grade ≥ 3.

**Interpretation:**

The results suggest that regardless of all proposed factors predictive for RP, the most important influenceable significant factor still is dose to the lung. The main aim should be to avoid RP grade ≥ 3, where a substantial proportion of patients suffer from prevailing sequalae. Consequently, the technical improvement and precision of radiotherapy delivery should continue to focus on lung sparing techniques also in the ongoing immunotherapy-containing schedules where the risk of pneumonitis may be increased.

## Introduction

For patients diagnosed with unresectable non-small cell lung cancer (NSCLC), chemoradiotherapy (CRT) is considered the standard treatment [1]. CRT may also be a treatment option in a subset of patients with stage II disease as well as for some patients with stage IV disease and solitary distant metastasis. The latest development in the management of stage III disease is the use of adjuvant PDL1 inhibition, which is now added after CRT in the regular treatment strategy for selected patients due to the results of the PACIFIC trial [2].

The most prominent side effects of CRT are oesophagitis and radiation pneumonitis (RP) where the latter is potentially fatal and harder to predict despite the use of modern dose constraints. RP is a non-infectious inflammation of the lung tissue that is manifested within the first 6 months after given treatment and RP is associated with an increased mortality risk [3, 4]. Pneumonitis is graded depending on severity, and commonly used grading systems include the Common Terminology Criteria for Adverse Events (CTC-AE) and Radiation Therapy Oncology Group (RTOG) [5]. Previous studies have reported that 10% – 40% of patients treated with CRT will experience RP of various grades [6–8].

There are numerous studies reporting on associations between patient-related and treatment-related factors and the risk of developing RP. It is well established that dose-volume parameters such as V_20Gy_ (percentage of the lung volume receiving > 20 Gy) and mean lung dose (MLD) are predictive factors for RP [6, 7, 9, 10]. In addition, there are several other treatment-related factors suggested to potentially be associated with RP, such as prescribed dose and fractionation, radiation treatment technique, and choice of chemotherapy agent [7, 9, 11, 12]. With regard to patient-related factors, previous research has suggested associations with factors such as age, smoking status, performance status and pre-radiotherapeutic lung function [6, 11–14].

To assess the risk for a patient to develop RP, a combination of patient- and treatment-related factors may have a better predictive value than the use of a single factor [14], and in a meta-analysis [6], the significant predictors of symptomatic RP were V_20Gy_ and chemotherapy with carboplatin/paclitaxel. Despite the current knowledge, there is still no clear consensus regarding which predictive factors that are the most important, and there is limited data on the ability of patients to recover from RP and the risk of developing chronic symptoms. To further elucidate on these uncertainties and help in clinical decision making, this study aims to analyse RP frequencies, rate of recovery and remaining sequelae and to assess the most important predictive factors for developing RP.

## Materials and methods

### Study design

We conducted a longitudinal, retrospective population-based cohort study of all consecutive patients in western Sweden with histologically or cytologically verified NSCLC treated with CRT from a single coordinating clinic between 2002 and 2016. Approval was granted by the Regional Ethical Review Board in Gothenburg, Sweden. Patients were eligible if they received CRT with curative intent, and the treatment was given with conventional fractionation or hyperfractionation for a delivered total dose of ≥ 56 Gy. The main exclusion criteria were death from causes other than RP within 180 days from end of treatment and patients not eligible for chemotherapy. The reason for excluding patients who died from causes unrelated to RP within 180 days of completing radiation treatment was to avoid uncertainties in the model analysis. To include these patients, they would need to be categorised either as having RP or not. Since this information was unavailable, their inclusion would have introduced ambiguity.

Potentially eligible patients and treatment-related data were identified and extracted from the Oncology Information System (OIS) at the radiotherapy department. Patient’s data were collected from the national lung cancer registry and patient charts.

Symptomatic RP (grade ≥ 2) was assessed retrospectively by two physicians through electronic medical records, scrutinising the initial assessment and computed tomography scans with access to prescription of, for example, corticosteroids and graded according to CTC-AE version 4.0 from the end of radiation therapy treatment until 180 days.

The assessments of potential predictive RP factors were performed on all patients with symptomatic RP (grade ≥ 2) and severe RP (grade ≥ 3) separately.

The rate of recovery and prevailing sequelae regarding respiratory symptoms, such as dyspnoea and cough after RP treatment, was obtained retrospectively through evaluation of patient charts using CTC-AE v. 4.0. The assessment was based on standard follow-up appointments at one-, and hereafter every third month up to 1 year after completion of radiotherapy. Prevailing sequelae were defined as remaining symptoms ≥ 3 months after RP treatment and residual symptoms (≥ 6 months) resulting in prolonged corticoid steroid treatment or persisting symptoms at the time of death.

### Variable selection

We considered variables previously reported to be important in RP and extracted data with regard to MLD, V_20Gy_, dose to 10% and 20% of the lung volume (i.e., D_10%_ and D_20%_, respectively), chemotherapy agent (platinum and vinorelbine, platinum and taxane, or others), administration method (concurrent or sequential), average radiation fractions per week (grouped according to >5.5 fractions/week given with 2 Gy per fraction, ≈ 10 fractions/week given with 1,7 Gy BID, or ≤ 5.5 fractions/week given with 2 Gy per fraction), prescribed dose, radiation treatment technique (three-dimensional conformal radiotherapy – 3DCRT, vs. intensity modulated radiotherapy – IMRT or volumetric-modulated arc therapy – VMAT), forced expiratory volume in the first second (FEV_1_), smoking status (a former smoker was defined as having quit smoking >1 year ago), histology, stage, performance status (PS), body mass index (BMI), age at the start of radiation therapy (RT), sex and tumour location.

To increase the stability of the final models, we reduced the number of variables [15]. The aim was to meet the rule-of-thumb of 10 events per variable. The variables were selected according to clinical prioritisation. It was also required that each subcategory of the categorical variables had at least two events and that the selected variables did not correlate with any other variable. Linear correlations between continuous variables were investigated by calculating the Pearson correlation coefficient, and variables with correlation coefficients above 0.7 were considered to be correlated with one another, and categorical variables were investigated by creating tables of two variables at a time and studying their patterns.

### Statistical analysis

We used imputation to handle missing data. For continuous variables, we used mean substitution of the total patient group, and for the categorical variables, we substituted with the most common subcategory.

For the two endpoints (RP grade ≥ 2 and grade ≥ 3), univariable logistic regression was performed for each selected variable. The odds ratios (ORs) and the corresponding 95% confidence intervals (CIs) were estimated. Variables with *p*-values less than 0.05 in likelihood ratio tests were considered statistically significant. Multivariable analysis using stepwise logistic regression with backwards elimination and Akaike information criterion (AIC) as the criterion was performed on the global model including all selected variables. ORs and their 95% CIs were calculated for all variables in the final multivariable model. The area under the receiver operating characteristic (ROC) curve was calculated.

Stability investigations were performed for both endpoints by repeating the stepwise regression procedure on 1000 bootstrapped datasets generated using random sampling with replacement. The bootstrap inclusion frequency, root mean squared deviance (RMSD) ratio and relative conditional bias were calculated for each variable [15]. The median of the bootstrapped regression coefficients and the 2.5th and 97.5th percentiles were calculated as well. All analyses were performed in RStudio (Version 1.2.5042, RStudio, Inc., Boston, MA, USA) using R (Version 4.0.2, R Core Team, Vienna, Austria).

The frequencies of RP grades and rate of recovery are reported descriptively.

## Results

**Figure 1:**
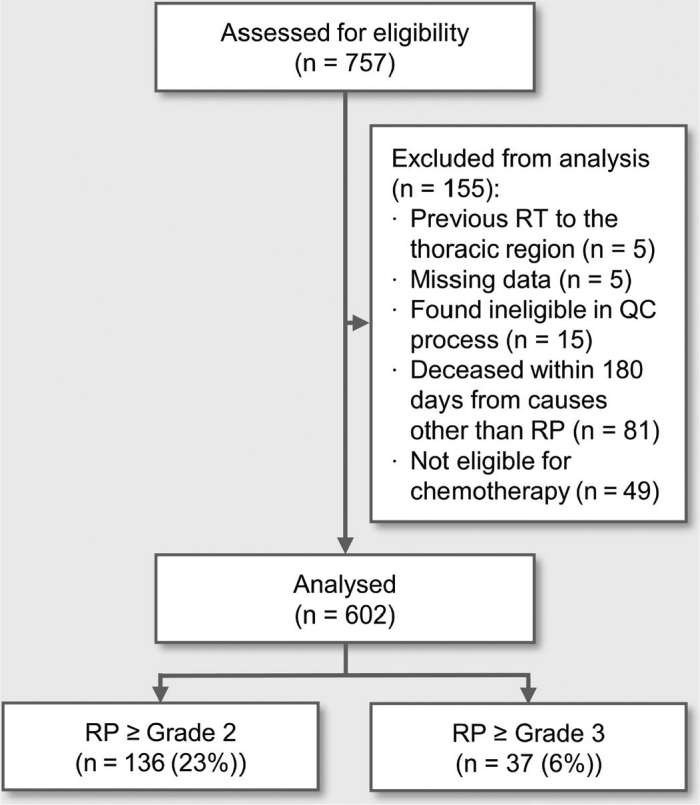
Study flow chart of patient eligibility. RT: Radiation therapy; QC process: quality control process; RP: Radiation pneumonitis.

The screening resulted in 757 patients. Of these, 155 patients were excluded because they did not meet the eligibility criteria (Figure 1).

### Patient, tumour, and treatment characteristics

A total of 602 patients were included and eligible for assessment of RP frequency, rate of recovery and potential predictive factors. The details of the patient, tumour and treatment-related characteristics are described in Table 1. In short there was an even distribution between sexes, and the vast majority of patients had a PS of 0–1. Approximately 90% were active or former smokers and the majority had stage III disease. All patients received full-dose radiotherapy with a dose between 56.0 and 84.0 Gy.

**Table 1:** Patient and treatment characteristics.

Variables	Number of patientsunless otherwise stated (%)
**Age at start of RT [years], median (range)**	66.9 (37.2–86.3)
Sex	
Male	306 (50.8)
Female	296 (49.2)
**Performance status**	
0	190 (31.6)
1	369 (61.3)
2	43 (7.1)
**BMI [kg/m^2^], median (range)**	24.4 (15.1–46.6)
**FEV1 [L], median (range))**	2.0 (0.6–4.7)
**Smoking status**	
Smoker	290 (48.2)
Never smoker	52 (8.6)
Former smoker	260 (43.2)
**Histology**	
Adenocarcinoma	300 (49.8)
Squamous Cell Carcinoma	225 (37.4)
NSCLC NOS	72 (12.0)
Other	5 (0.8)
**Tumour location**	
Upper lobe	323 (53.7)
Main bronchus	41 (6.8)
Multifocal	8 (1.3)
Middle lobe	31 (5.1)
Lower lobe	199 (33.1)
**Stage grouping**	
IA	17 (2.8)
IB	68 (11.3)
IIA	18 (3.0)
IIB	44 (7.3)
IIIA	198 (32.9)
IIIB	229 (38.0)
IV	28 (4.7)
**Chemotherapy agents**	
Platinum-Vinorelbine	441 (73.3)
Platinum-Taxane	143 (23.7)
Other	18 (3.0)
**Chemotherapy administration**	
Concurrent	560 (93)
Sequential	42 (7)
**Radiotherapy technique**	
3DCRT	551 (91.5)
VMAT	46 (7.6)
IMRT	5 (0.8)
**Prescribed dose**	
60 Gy	66 (11)
64.6 Gy	247 (41.0)
66 Gy	5 (0.8)
68 Gy	29 (4.8)
70 Gy	245 (40.7)
Other	10 (1.7)
**Average fractions/week**	
>5.5	251 (41.7)
≈ 10	249 (41.4)
≤ 5.5	102 (16.9)
**V_20 Gy_[%], median (range)**	26.7 (0–60.1)

BMI: Body mass index; FEV1: Forced expiratory volume in one second; NSCLC NOS: Non-small-cell *lung cancer -not otherwise specified*; 3DCRT: Three-dimensional conformal radiotherapy; IMRT: intensity modulated radiotherapy; VMAT: volumetric-modulated arc therapy; V_20 Gy_ [%]: Relative volume receiving at least 20 Gy.

### Frequency of radiation pneumonitis

The median time to the development of symptomatic RP was 64 days (range 8–179) after the end of RT. A total of 136 (23%) patients developed RP grade ≥ 2 and 37 (6%) developed RP grade ≥ 3, and divided per grade, 99 (16%) patients developed RP grade 2, 24 (4%) grade 3, no patients developed grade 4, and 13 (2%) grade 5.

### Selection and modelling of predictive factors for radiation pneumonitis

The continuous variables MLD, V_20Gy_, D_10%_ and D2_0%_ were correlated, as were the categorical variables average fractions per week and treatment technique. Therefore, V_20Gy_ and average fractions per week were selected for use in the analysis. Missing FEV1 (15 patients) and BMI (5 patients) data were replaced with the respective mean values of the total population. Univariable analyses of potential predictive factors for the development of RP grade ≥ 2 and grade ≥ 3 are presented in Table A.1 and A.2 in Supplementary Appendix 1.

In the multivariable analysis, the selected model for predicting RP grade ≥ 2 included V_20Gy_, smoking status, average fractions per week and chemotherapy agent (Table 2). The selected model for RP grade ≥ 3 included V_20Gy_ and age at start of RT (Table 2).

**Table 2:** The final models of stepwise multivariable analyses predicting radiation pneumonitis grade ≥ 2 and grade ≥ 3, respectively.

	RP grade ≥ 2				RP grade ≥ 3			
	β	OR	95% CI	*p*	β	OR	95% CI	*p*
Intercept	−2.73			< 0.001	−10.56			< 0.001
**Age [years]**	-	-	-	-	0.08	1.09	[1.04 – 1.14]	< 0.001
**V_20 Gy_ [%]**	0.05	1.05	[1.03 – 1.07]	< 0.001	0.07	1.07	[1.03 – 1.11]	< 0.001
**Smoking status**					-	-	-	-
Smoker	*Reference*							
Non-smoker	0.37	1.44	[0.68 – 2.91]	0.316				
Former smoker	0.64	1.90	[1.25 – 2.9]	0.003				
**Chemotherapy agent**					-	-	-	-
Platinum + Vinorelbine	*Reference*							
Platinum + Taxane/Other	−0.40	0.67	[0.41 – 1.08]	0.108				
**Average fractions/week**					-	-	-	-
> 5.5	*Reference*							
≈ 10	−0.44	0.65	[0.42 – 1]	0.050				
≤ 5.5	−0.32	0.73	[0.39 – 1.31]	0.300				

RP: Radiation pneumonitis; Regression coefficient; OR: Odds ratio; CI: Confidence interval; V_20 Gy_ [%] = Relative volume receiving at least 20 Gy.

**Figure 2:**
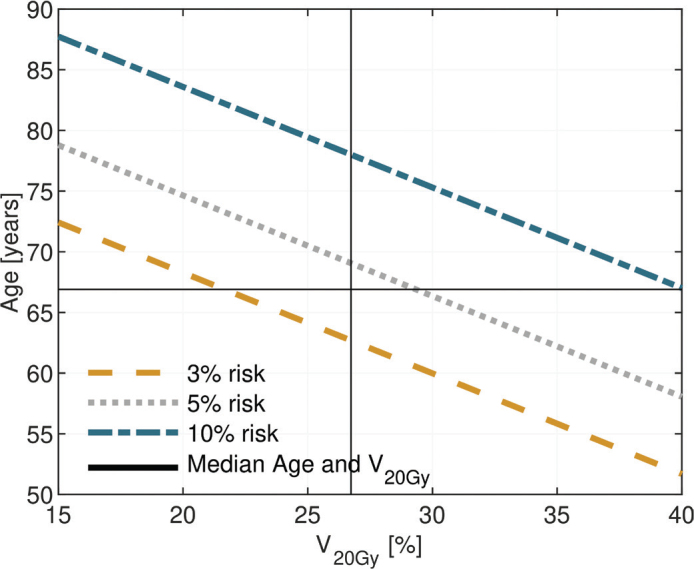
Iso-probability curves of the risk of radiation pneumonitis grade ≥ 3 as a function of age and V_20Gy_. V_20 Gy_ [%] = Relative volume receiving at least 20 Gy.

To exemplify the risk of RP grade ≥ 3 estimated by the model, iso-probability curves in Figure 2 illustrate combinations of V_20Gy_ and age associated with a risk of 3%, 5% and 10%.

**Figure 3:**
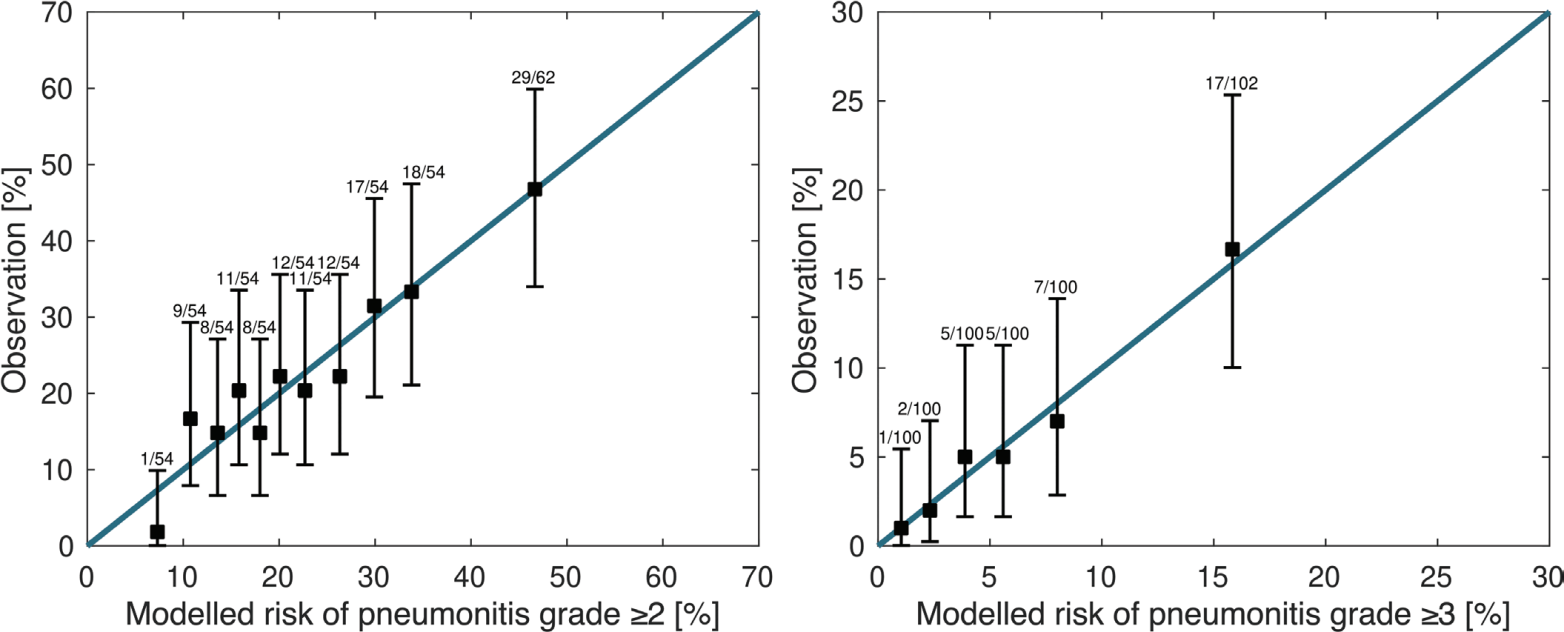
Calibration plots for radiation pneumonitis grade ≥ 2 and grade ≥ 3 endpoints.

ROC analysis resulted in an area under the curve (AUC) of 0.67 for the endpoints RP grade ≥ 2 and 0.74 for RP grade ≥ 3. Calibration plots presented in Figure 3 show the agreement between actual risk and predicted risk according to the final multivariable model. The results of the stability investigations can be found in the Supplemental Appendix (Table B.1 and Table B.2).

### Rate of recovery from radiation pneumonitis

A total of 123 (20%) patients developed symptomatic RP grade 2–4, 29 (24%) patients were not eligible for evaluation of recovery rate due to confounding factors such as overlapping symptoms or increased use of corticoid steroids for reasons other than progressive RP or due to missing data.

Out of the 94 evaluable patients with RP grade 2–4, 27 (29%) patients suffered with prevailing sequela. A total of 12% (9 out of 73) patients were chronically afflicted in the RP grade 2 group and 86% (18 out of 21) among patients with RP grade 3. In general, there were more severe sequelae in the RP grade 3 group than in patients with RP grade 2. The rates of prevailing sequelae after RP grade 2 and grade 3 are illustrated in Table 3.

**Table 3:** Patients with prevailing sequelae affecting activity of daily living.

Grade of pneumonitis	Total no. of patients with prevailing sequela	Individual symptoms in patients with prevailing sequalae		
		No. of patients with dyspnoea	No. of patients with cough	No. of patients on long-term oxygen therapy
**RP grade 2** *n* = 99 (Assessable *n* =73)	9 (12.3%) Missing data 6 (6.1%) Not assessable 20 (20.2%)	Grade 2: 6 (8.2%)	Grade 2: 4 (5.5%)	0 (0%)
		Grade 3: 1 (1.4%)		
**RP grade 3** *n* = 24 (Assessable *n* = 21)	18 (85.7%) Missing data 1 (4.2%) Not assessable 2 (8.3%)	Grade 2: 8 (38.1%)	Grade 2: 3 (14.3%)	11 (52.4%)
		Grade 3: 9 (42.9%)	Grade 3: 1 (4.8%)	

RP: Radiation pneumonitis.

## Discussion

Several potential predictive factors of RP have been postulated throughout the years, and here, in one of the largest consecutive population-based studies we found that the main modifiable factor that still should be our main focus is the dose to the lung. Most other factors are of less importance or nonmodifiable, with the exception of being cautious with older patients. The aim is to decrease the risk of RP grade 3 as those patients have a very high risk of developing remaining sequelae.

The overall symptomatic RP frequency was 23%, with a median time to onset of 64 days. These findings are consistent with previous results indicating an RP incidence ranging from 10 to 40% starting between 40 and 80 days after treatment completion in patients with NSCLC treated with CRT. RP frequencies differ substantially between studies, but the most likely interval is elucidated in a meta-analysis [6, 7, 13–19].

Most patients with symptomatic RP developed a grade 2 reaction (16% of the total population), whereas another 6% of the patients developed more severe toxicity (i.e., RP grade ≥ 3). Previous reports on severe RP are in line with our results and range between 4 and 12% [8, 13, 14, 18, 20, 21]. Notably, we did not observe any patients with RP grade 4, similar to previous studies reporting no/or small numbers (~ 0–3%) of RP grade 4 [7, 13, 14, 18]. Patients disproportionally more often acquired fatal RP in comparison to grade 4, a finding also reported in meta-analyses [6, 21]. This may be attributed to a threshold being reached with life-threatening extensive severe inflammation in need of intensive care (grade 4) being very hard to reverse with a high mortality risk.

When analysing potential predictive factors for symptomatic RP, the selected model included V2_0Gy_, smoking status, average fractions per week and chemotherapy agent as predictive of RP grade ≥ 2, whereas for RP grade ≥ 3, V_20Gy_ and age at start of RT were selected factors. The fact that ongoing smoking may be a protective factor against the development of RP was demonstrated by Hernando et al. [18] and in the meta-analysis by Vogelius et al. [12] in 2012. Non-smokers have a higher risk than on-going smokers but a lower risk than former smokers. Although there is a clear association between smoking and a decreased risk of RP, there is no real applicability in clinical practice, as all patients are encouraged to smoke cessation.

The majority of patients in this study received platinum combined with vinorelbine in their CRT schedule. These results imply an increased risk of RP grade ≥ 2 with this regimen compared to platinum combined with taxane. In contrast, previous studies, such as by Dang et al. [19] in 2014, demonstrated a greater risk of severe RP in patients receiving concurrent cisplatin and docetaxel compared to cisplatin and vinorelbine. In addition, there are reports showing no correlation between chemotherapy agents and the risk of developing symptomatic RP [14, 16]. In the present cohort the choice of chemotherapy depended on current standard that changed over time and not on patient factors such as age or performance status. Overall, with conflicting data and chemotherapy agents being the least important among the investigated factors in our model, we consider it to be of little importance in the daily RP risk assessment.

The model in our work also included the fractionation schedule (i.e., average fractions per week) as predictive of RP grade ≥ 2, favouring ≈ 10 fractions/week relative to > 5.5 fractions/week. This result implies that hyperfractionated RT would be preferable. However, it is difficult to draw any conclusions from this result, as the average fractions per week was mildly correlated with treatment technique, prescribed dose and to treatment period, as clinical practice changed over time.

Age as a predictive factor for RP has been demonstrated in several studies where Dang et al., among others, demonstrated an association with age for both RP grade ≥ 2 and RP grade ≥ 3 [19, 22]. The latter is also described by Tsujino et al., with a significant increased risk of severe RP for patients ≥ 68 years [14]. According to our study, age combined with V_20Gy_ were significant predictors for RP grade ≥ 3. Iso-probability curves in Figure 2 illustrates how one may translate these data into a clinical context for a risk assessment of severe RP, where higher age and dose to the lung should be considered in treatment decisions.

Several factors related to lung dosimetry were correlated, which is why only V_20Gy_ out of these factors was considered for further analysis. Several studies have demonstrated that different dosimetric lung variables, such as V2_0Gy_, MLD and V_10%_, are of significance for the risk of developing RP [4, 14, 18, 19]. However, as reported in this study and stated by Palma et al. [6], dosimetric data tend to be collinear, and differences in predictive value may be minimal and unlikely to be of clinical importance. According to the model, dose to the lung is the most important modifiable factor and is still meaningful to reduce despite being below accepted constraint levels. Several other clinical factors, such as performance status, tumour location, weight loss and pretherapeutic lung function, have been suggested as predictive factors for RP [7, 13, 14, 18]. In this study, we did not observe such associations, and given this rather large cohort, it is reasonable to assume that after the initial selection of patients suitable for CRT (based on, e.g., PS and pretherapeutic FEV_1_), these factors are not of major importance.

When analysing recovery rate and prevailing sequelae, we observed that a majority of the evaluable patients (*n* = 67 (71%)) who suffered from symptomatic RP recovered after corticosteroid treatment, but with a markedly worse recovery rate among patients with RP grade 3 compared to RP grade 2 (86% and 12% with prevailing sequalae, respectively). A similar result was also described by Graham et al., where 70% of the patients with RP grade 2 after CRT recovered, while none of the patients suffering from RP grade 3 regained function [17]. Overall, the recovery rate and prevailing sequelae after RP are underexplored and need to be further addressed. However, the main aim should be to prevent the development of RP grade ≥ 3 due to the low recovery rate, high risk of prevailing sequalae, and increased risk of mortality.

In this study, we assessed RP before the addition of immunotherapy to standard CRT treatment. In previous clinical trials, there was no clear signal of an increased risk of pneumonitis with the addition of PDL1 inhibition after CRT. Two other studies however indicate lower cut-off levels for increased risk of symptomatic pneumonitis than pre-existing dose constraint of V_20Gy_ < 35% [23, 24]. If these data can be reproduced in future studies, they might result in a need for stricter constraints for lung tolerance when adding PDL1 inhibition.

This study used a retrospective design with associated limitations. The RP diagnosis and prevailing sequelae was based on medical records where assessment and grading at a later time point then when the RP manifested itself may introduce uncertainties. A few medical records were deficient with respect to the degree of symptom severity, leaving interpretation to the analysist, which increases the risk of bias and subjective assessment. However, CTC-AE takes medical intervention into consideration for grading RP ≥ 2, and often oxygen use for RP grade 3, and combining the assessment of medical records, together with radiographic images, laboratory test results and prescription records strengthens the classification of symptomatic RP. In addition, when analysing records over a 15-year period, changes in documentation and methodology i.e. type of chemotherapy or radiotherapy technique, occurred.

## Conclusion

In this study we observed an incidence of symptomatic RP in approximately one-fourth of patients with locally advanced NSCLC treated with CRT. Patients who developed severe RP had a substantially increased risk of chronic symptoms despite adequate medical treatment. The aim of RP risk assessments should therefore, in particular, be to decrease the risk of RP grade ≥ 3, and the most important factors to take into clinical consideration in this respect are dose to the lung and age. Most other proposed factors contributing to RP are likely of less clinical importance, and the focus in management ought to strive for technical improvement and precision of radiotherapy delivery with lung-sparing techniques, also in the ongoing immunotherapy-containing schedules where the risk of pneumonitis may be increased.

## Supplementary Material

Assessment of radiation pneumonitis and predictive factors in patients with locally advanced non-small cell lung cancer treated with chemoradiotherapy

## Data Availability

Research data are stored in an institutional repository and will be shared upon request to the corresponding author.
