# Supplementary material for: Assessment of radiation pneumonitis and predictive factors in patients with locally advanced non-small cell lung cancer treated with chemoradiotherapy
Source: Acta Oncol. 2024 Oct 16;63:40576. doi: 10.2340/1651-226X.2024.40576 (PMC11495145; doi:10.2340/1651-226X.2024.40576)
Supplement: Assessment of radiation pneumonitis and predictive factors in patients with locally advanced non-small cell lung cancer treated with chemoradiotherapy [file AO-63-40576-s1.pdf]

Supplementary material has been published as submitted. It has not been copyedited, or typeset by Acta Oncologica

Table A.1 Univariable analysis of potential predictive factors for radiation pneumonitis grade ≥2

Variables	β	OR	95% CI	p value
**V20 Gy [%]**	0.05	1.05	[1.03-1.07]	<0,001
**Chemotherapy agent**				
Platinum + Vinorelbine	Reference			
Platinum + Taxane/Other	-0.22	0.80	[0.51-1.24]	0.34
**Average fractions/week**				
> 5.5	Reference			
≈ 10	-0.54	0.59	[0.38-0.89]	0.01
≤ 5.5	-0.46	0.63	[0.35-1.09]	0.11
**FEV1 [L]**	0.19	1.21	[0.93-1.58]	0.15
**Smoking status**				
Smoker	Reference			
Never smoker	0.47	1.60	[0.77-3.15]	0.19
Former smoker	0.63	1.87	[1.25-2.83]	0.003
**Performance status**				
0	0.25	1.28	[0.85-1.93]	0.24
1	Reference			
2	0.27	1.30	[0.6-2.63]	0.48
**BMI [kg/m^2]**	0.04	1.04	[1-1.09]	0.07
**Age at start of RT [years]**	0.02	1.02	[1-1.04]	0.11
**Sex**				
Male	Reference			
Female	-0.19	0.83	[0.57-1.22]	0.34
**Tumour location**				
Upper lobe	Reference			
Main bronchus/Multifocal/Middle	0.05	1.05	[0.56-1.88]	0.87
Lower lobe	0.35	1.41	[0.93-2.14]	0.10

Abbreviations: β = Regression coefficient, OR = Odds ratio, CI = Confidence interval, $V_{20\,Gy}$ [%] = Relative volume receiving at least 20 Gy, FEV1 [L] = Absolute forced expiratory volume in the first second, BMI = Body mass index, RT = Radiation therapy.

Table A.2 Univariable analysis of potential predictive factors for radiation pneumonitis grade ≥3

Variables	β	OR	95% CI	p value
**V20 Gy [%]**	0.05	1.06	[1.02-1.09]	0.001
**Average fractions/week**				
> 5.5	Reference			
≈ 10	-0.36	0.70	[0.32-1.48]	0.35
≤ 5.5	0.16	1.17	[0.46-2.73]	0.72
**FEV1 [L]**	-0.30	0.74	[0.44-1.19]	0.23
**Smoking status**				
Smoker	Reference			
Former/Never smoker	0.33	1.39	[0.71-2.79]	0.34
**Age at start of RT [years]**	0.07	1.07	[1.03-1.12]	0.002

Abbreviations: β = Regression coefficient, OR = Odds ratio, CI = Confidence interval, $V_{20\ Gy}$ [%] = Relative volume receiving at least 20 Gy, FEV1 [L] = Absolute forced expiratory volume in the first second, RT = Radiation therapy.

Table B.1 Radiation pneumonitis grade ≥ 2 – Stability investigation

	Global model		Final model		Bootstrapping					
**Variables**	**β**	**SE**	**β**	**SE**	**Bootstrap inclusion frequency [%]**	**RMSD ratio**	**Relative conditional bias [%]**	**β median**	**2.5th percentile**	**97.5th percentile**
(Intercept)	-4.90	1.39	-2.72	0.36	100	1.09	-3.4	-4.62	-7.92	-2.31
V20 Gy [%]	0.05	0.01	0.05	0.01	100	1.09	4.4	0.05	0.03	0.08
Smoking status										
Smoker	Reference		Reference					Reference		
Never smoker	0.24	0.38	0.37	0.37	72.4	1.08	14.9	0.10	-0.65	1.05
Former smoker	0.50	0.23	0.64	0.22	72.4	1.46	27.7	0.54	0.00	1.01
Average fractions/week										
> 5.5	Reference		Reference					Reference		
≈ 10	-0.40	0.23	-0.44	0.22	59.8	1.38	43.3	-0.44	-0.90	0.00
≤ 5.5	-0.28	0.31	-0.32	0.31	59.8	1.10	58.2	-0.08	-0.98	0.16
Age at start of radiation therapy [years]	0.02	0.01			53.7	1.19	78.0	0.02	0.00	0.05
Chemotherapy agent										
Platinum+ Vinorelbine	Reference							Reference		
Platinum+ Taxane/Other	-0.33	0.26	-0.40	0.25	49.8	1.30	83.3	0.00	-0.97	0.00
BMI [kg/m2]	0.03	0.02			48.6	1.24	70.9	0.00	0.00	0.08
FEV1 [L]	0.11	0.19			29.9	0.93	175.3	0.00	0.00	0.50
Sex										
Male	Reference							Reference		
Female	-0.11	0.25			25.9	0.86	205.7	0.00	-0.61	0.31
Tumour location										
Upper lobe	Reference							Reference		
Main bronchus, Multifocal or Middle lobe	0.04	0.33			24.2	0.82	-131.5	0.00	-0.75	0.63
Lower lobe	0.19	0.23			24.2	1.07	94.0	0.00	-0.08	0.67

Performance status										
0	0.11	0.23			19.8	0.82	208.7	0.00	-0.01	0.57
1	Reference							Reference		
2	0.18	0.39			19.8	0.84	102.6	0.00	-0.42	0.93

Abbreviations: β = Regression coefficient, SE = Standard error, RMSD = Root mean square deviation, V20 Gy [%] = Relative volume receiving at least 20 Gy, BMI = Body mass index and FEV1 [L] = Absolute forced expiratory volume in the first second.

Table B.2 Radiation pneumonitis grade ≥ 3 – Stability investigation

	Global model		Final model		Bootstrapping					
**Variables**	**β**	**SE**	**β**	**SE**	**Bootstrap inclusion frequency [%]**	**RMSD ratio**	**Relative conditional bias [%]**	**β median**	**2.5th percentile**	**97.5th percentile**
(Intercept)	-9.76	2.12	-10.56	1.87	100	1.07	6.8	-10.52	-14.47	-5.91
$V_{20\ Gy}$ [%]	0.07	0.02	0.07	0.02	99.5	1.11	3.9	0.07	0.03	0.11
Age	0.08	0.02	0.08	0.02	99.2	0.93	8.1	0.08	0.04	0.12
FEV1 [L]	-0.24	0.29			30.4	1.11	131.7	0.00	-0.89	0.00
Average fractions/week										
> 5.5	Reference							Reference		
≈ 10	-0.11	0.40			27.6	0.79	98.4	0.00	-0.93	0.57
≤ 5.5	0.34	0.46			27.6	0.99	120.0	0.00	0.00	1.30
Smoking status										
Smoker	Reference							Reference		
Former/Never smoker	0.11	0.36			18.7	0.92	187.0	0.00	-0.68	0.91

Abbreviations: β = Regression coefficient, SE = Standard error, RMSD = Root mean square deviation, V20 Gy [%] = Relative volume receiving at least 20 Gy, BMI = Body mass index and FEV1 [L] = Absolute forced expiratory volume in the first second.
